# An Investigation Into Interpersonal and Peripersonal Spaces of Chinese People for Different Directions and Genders

**DOI:** 10.3389/fpsyg.2020.00981

**Published:** 2020-06-05

**Authors:** Xiaoqing Yu, Wei Xiong, Yu-Chi Lee

**Affiliations:** School of Design, South China University of Technology, Guangzhou, China

**Keywords:** interpersonal space, peripersonal space, spatial judgment, direction, gender difference

## Abstract

This study explores the interpersonal space (IPS) and peripersonal space (PPS) of Chinese people and evaluates the relationship between the two spaces for different directions and genders. Seventy-one participants were recruited for this study. Participants were required to determine their IPS in eight directions (0°, 45°, 90°, 135°, 180°, 225°, 270°, 315°) when approached by male or female confederates in the comfort distance task. Each participant was also asked to judge their PPS in five directions (0°, 45°, 90°, 270°, 315°) following the same procedure. Results showed that their IPS was significantly influenced by direction (*p* < 0.05), with the largest distance in the front (0°) and the closest distance in the rear (135°, 180°, 225°), indicating non-circular IPS among Chinese subjects. Moreover, the PPS on the right side (90°) was larger than in other directions (0°, 45°, 270°, 315°). Participants maintained larger IPS than PPS in the front, but the IPS was closer than PPS on the right and left sides. When facing a female confederate, larger IPS was preferred than PPS, whereas the opposite held true when facing a male confederate. Comparison of participants’ arm length and PPS showed that the reachability distance was overestimated in the front but underestimated laterally. The findings of this study can be applied to environmental design, space utilization, and social interaction.

## Introduction

The space surrounding individuals is an important area because it is where people interact with social stimuli in the external environment. Social psychology and neurocognitive research has focused on the space around our bodies, indicating that there is a particular relationship between the interpersonal social space (comfort distance) and peripersonal action space (reachability distance) (Iachini et al., 2015; Ruggiero et al., 2017; Cartaud et al., 2018; D’Angelo et al., 2019; Spaccasassi et al., 2019). Interpersonal space (IPS) is defined by social psychology as a safety buffer zone that individuals maintain between themselves and others (Hall, 1966; Sommer, 1969; Hayduk, 1983). Intrusion into the IPS range could generate discomfort and arousal. People tend to keep a larger interpersonal distance from intruders in uncomfortable situations (Hayduk, 1983; Linkenauger et al., 2009; Holt et al., 2014). The typical method for measuring IPS is based on comfort distance judgments in which participants stop a confederate’s approach when they were comfortable but on the point of being uncomfortable (Hayduk, 1983; Adams and Zuckerman, 1991; Nandrino et al., 2017).

In the neurocognitive domain, this space is referred to as the peripersonal space (PPS). The PPS is represented by multisensory neurons including tactile, visual, and auditory stimuli near human bodies (Rizzolatti et al., 1981; Makin et al., 2007; Serino et al., 2011). Peripersonal space has been regarded as the interface where individuals can detect or predict interactions between themselves and the external environment (Colby, 1998; Grefkes and Fink, 2005). Previous studies demonstrated that the PPS boundary could be modulated by action manipulations, such as tool use (Farnè and Làdavas, 2000; De Vignemont and Iannetti, 2015). The reachable distance judgment was applied to determine the PPS size in several studies, where each participant was asked to estimate the reachable distance to a confederate (Delevoye-Turrell et al., 2011; Ruggiero et al., 2017). In addition, several studies have advanced the view that PPS is also affected by social interactions (Heed et al., 2010; Teneggi et al., 2013). In Teneggi et al. (2013), participants were asked to conduct a tactile detection task on their face while concurrent task-irrelevant sounds approached or receded from their faces. Peripersonal space was found to be larger when facing a mannequin than when facing another individual, indicating a link between sensorimotor processing and social cognition. De Vignemont and Iannetti (2015) suggested that the PPS could be interpreted as a goal-directed action area but also as a protective bubble. These two functions of the PPS require distinct sensory and motor processes.

Previous studies found the space surrounding individuals to be affected by many factors, such as culture, gender, age, and direction (Remland et al., 1995; Uzzell and Horne, 2006). In the theory of Hall (1966), the preferred social distance was most influenced by cultural norms. Early cross-cultural research on spatial behaviors reported that contact and non-contact cultures showed significant differences in IPS, and contact cultures preferred a closer distance (Hall, 1966; Baldassare and Feller, 1975). Sicorello et al. (2019) indicated that Japanese participants preferred overall larger IPS than German participants. Beaulieu (2004) evaluated the IPS of Anglo Saxons, Asians, Caucasians, Mediterraneans, and Latinos and found that Asians displayed the second largest comfort distance of all. Sorokowska et al. (2017) made a paper-and-pencil test to collect the preferred IPS from 42 countries, including China. The participants imagined that they were the people in the questionnaires, and only the direction in the front was elicited. Yu and Lee (2019) investigated the comfort distance of Chinese participants when they were asked to stand still. The results of the study indicated that the comfort distance of Chinese people in the front was significantly larger than in other directions. On the other hand, the PPS boundary has been evaluated in several different studies; however, the participants were all from Europe (Iachini et al., 2014, 2016; Nandrino et al., 2017; D’Angelo et al., 2019). Peripersonal space among the Chinese has not been investigated so far. Extensive investigation into IPS and PPS in Chinese culture in the actual environment is thus merited.

The IPS in different directions has been examined in several studies. Hayduk (1981) demonstrated that the shape of the IPS is non-circular, being slightly larger in the front than in the rear. Bailenson et al. (2003) conducted an IPS experiment in a virtual reality environment in which participants took a memory task when approaching a virtual agent from the front and rear. The results were consistent with the findings of Hayduk (1981). In contrast, participants in Hecht et al. (2019) were asked to approach another participant from different directions and stop at a distance for a comfortable conversation. The results showed that the IPS presented no significant difference in eight directions and thus was circular. Currently, there has been little discussion of the IPS among Chinese people in different directions. Some studies of the PPS concentrated only on the distance in the front direction (Ruggiero et al., 2017, 2019; D’Angelo et al., 2019). The PPS boundary in different directions also remains unclear.

Research on gender differences in the IPS is quite abundant. Several studies found that male dyads maintained the largest comfort distance and female dyads maintained the closest distance, whereas mixed-gender dyads maintained an intermediate distance (Caplan and Goldman, 1981; Aliakbari et al., 2011; Hecht et al., 2019). In other studies, the closest distance was found in mixed-gender dyads, followed by female dyads and male dyads (Baxter, 1970; Evans and Howard, 1973). White (1975) reported that female participants kept a closer distance to the confederate than male ones, but no significant effect was found of the confederate’s gender. Uzzell and Horne (2006) indicated that the gender role showed a significant main effect on IPS, but no significant effect appeared for biological sex and sexuality. Only a few studies investigated the gender difference in the PPS (Iachini et al., 2014, 2016; D’Angelo et al., 2019). There have been few detailed studies of gender differences in the IPS and PPS of Chinese participants.

Previous studies discussed the relationships between IPS and PPS. Iachini et al. (2014) examined the two spaces in the front direction (0°) in a virtual reality environment. Results showed that IPS was larger than PPS when the participants were approached passively, but IPS and PPS were similar when participants actively approached the confederate. The findings indicate that the two spaces share a common motor nature to different degrees. The IPS and PPS are not only physically similar in size but also sensitive to social stimuli. Iachini et al. (2016) evaluated gender and age effects under real conditions and in a virtual reality environment, finding that both the IPS and PPS expanded when facing a male confederate, but were reduced with a female confederate. Furthermore, participants tended to maintain larger IPS and PPS with adults than with children. Ruggiero et al. (2017) investigated whether facial expressions affected the IPS and PPS. An increasing IPS and PPS distance was found when seeing an angry face than a neutral or happy face, indicating that the IPS and PPS expanded in a threatening situation. Note that previous studies compared only the two kinds of distances in the front, and most of these studies were carried out in Europe. There is little information available on the relationship between IPS and PPS in the Chinese population in different directions.

Overall, cultural differences are one of the main factors affecting IPS and PPS. China is classified as a non-contact culture (Hasler and Friedman, 2012). In daily life, the subways, bus stations, and other public facilities in China are usually very crowded because China has the largest population in the world. Thus, the body space boundary of Chinese people might be different under specific environments. There is little information available on the IPS and PPS of Chinese people and the differences between the two spaces in the Chinese population. Hence, this study investigates the IPS and PPS of Chinese people in different directions and evaluates the relationships between the two spaces, taking gender effects into consideration. Participants were requested to determine their comfort and reachability distances when they were approached from different directions. The experimental paradigm was devised based on that of Iachini et al. (2014). This study contributes to a deeper understanding of the IPS and PPS near human bodies and provides important insights into the motor nature of IPS and PPS in Chinese culture. The IPS and PPS range data could be applied to environmental design, space utilization, and social interaction.

## Materials and Methods

### Participants

Seventy-one participants (36 females), aged from 18 to 36 years old [mean = 21.0, standard deviation (SD) = 2.7], were recruited for this study. All participants were right-handed. Each participant had normal or corrected-to-normal vision. The average stature and body weight of male participants were 171.5 ± 6.1 cm and 60.3 ± 7.3 kg, respectively. The average stature and weight of female participants were 161.8 ± 4.2 cm and 53.2 ± 8.8 kg, respectively. The average arm length of males was 73.7 cm, whereas that of females was 68.2 cm. The average shoulder breadth and the bigonial width of all participants were 37.4 and 11.6 cm, respectively. None of them had cognitive impairment or other illnesses that might affect distance perception. This was confirmed by self-reporting by each participant. Participants had no prior knowledge of the scientific purpose of these experiments. Informed consent was obtained from each participant. The experimental procedures were approved by the Institutional Ethics Committee of South China University of Technology.

### Experimental Setting and Confederates

**FIGURE 1:**
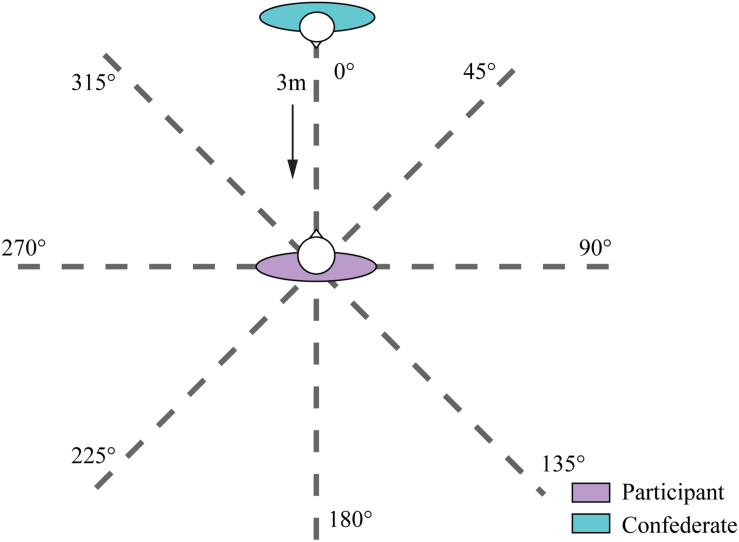
The experimental setting of this study.

These experiments were conducted in an empty room (4 m × 8 m × 4 m). Eight directions, equally spaced by 45° from 0° to 360°, were selected for evaluation. There were eight straight lines placed on the floor from a marked point to identify the eight approach directions for confederates. Participants were asked to stand upright with their feet shoulder-width apart on a marked point. A male and a female confederate with normal Chinese appearance were selected. The male and female confederates were 172.4 and 164.8 cm tall, respectively. Both confederates were unknown to all participants. During the experiment, confederates wore the same casual clothes without any accessories. Direct gaze has been found to generate a more intrusive response than averted gaze, and direct gaze could thus enlarge the interpersonal distance (Bailenson et al., 2003; Ioannou et al., 2014). In order to simulate a normal situation in daily life in the eight directions, confederates were required to maintain a neutral expression and maintain no eye contact with participants during each trial. The direction in front of participants was defined as 0°. Accordingly, the right, rear, and left sides of the participants were, respectively, defined as 90°, 180°, and 270°. The definitions of the eight directions around the participants are illustrated in Figure 1.

### Apparatus and Measurements

A digital laser measurer (JM-G25240; JIMIHOME, Shanghai, China) with an accuracy of 2 mm and 0.05–40 m measuring range was applied to measure the distance between the participant and the confederate. The digital laser measurer was calibrated before each experimental trial to ensure accuracy. When the confederate approached the participants in the front area (0°, 45°, 315°), the distance was measured from the confederate’s chin to the participant’s mental protuberance. When the participant was approached from the right (90°) or left side (270°), the distance was recorded between the confederate’s chin and the right or left mandibular angle points of each participant. In the rear directions (135°, 180°, 225°), the distance from the confederate’s chin to the participant’s fifth cervical vertebra was collected. These measure definitions were drawn from previous studies (Iachini et al., 2014, 2016).

### Procedure

Two tasks, comfort distance judgment (IPS) and reachability distance judgment (PPS), were conducted in this study. All experimental procedures were based on and modified from Iachini et al. (2014). Each participant received the experimental instructions and reported on their demographic data. The experimenter then described the experimental procedure orally to the participants. Before data collection, the arm length of each participant was measured for further analysis. In order to increase the accuracy and reliability of the distance measurements, four markers were attached to the surface of the participant’s mental protuberance, right and left mandibular angle points, and the fifth cervical vertebra.

For the comfort distance judgment task, participants were required to practice twice to familiarize themselves with the distance judgment. The participants were asked to stand at a point marked on the ground with their arms naturally at their sides. The initial distance between the participant and the confederate was 3 m. Either the male or female confederate approached the participants at a speed of 0.5 m per second from each of the eight directions. Participants were allowed to move their eyes to gauge the proximity of the confederate and were instructed to say “stop” at the moment they still felt comfortable but were about to feel uncomfortable due to the confederate’s approach. The confederate stopped immediately when the participant spoke out. All participants had a chance to slightly adjust the confederate’s position to reconfirm the comfort distance. The distance between the participant and the confederate was then collected by digital laser measurer. Each trial was repeated three times. The procedure was repeated for the male and female confederate in all eight directions. The 48 (8 directions × 2 confederates × 3 repetitions) trials were assigned in randomly order.

For the reachability distance judgment task, participants were instructed to raise their arms to feel the reachable distance around them (Linkenauger et al., 2009; Quesque et al., 2017). Similarly, all participants were asked to practice twice to familiarize themselves with the reachability distance judgment process. The male or female confederate walked toward the participants from one of five directions (0°, 45°, 90°, 270°, 315°) selected because PPS is represented primarily by visual, somatosensory, and proprioceptive modalities. The brain computes the positions of objects around the body through vision and judges whether they can be touched and manipulated with the arms through ontology perception (Holmes and Spence, 2004). A confederate approaching from behind is imperceptible via vision, so it is difficult for the participants to properly judge the PPS behind themselves. When people intend to grasp and manipulate objects behind them, they have to turn around and stand in front of the objects for better operation. Hence, only the distances in the front five directions were collected. Participants were instructed to say “stop” as soon as they felt they could touch the confederate. Participants also had the chance to slightly adjust the distance they chose. The distance between the confederate and participant was measured in the same way as the comfort distance judgment experiment. Each trial was repeated three times to ensure data quality. The procedure was repeated for the male and female confederates in the five directions. A total of 30 trials (5 directions × 2 confederates × 3 repetitions) were conducted and randomly assigned.

Each participant conducted a total of 78 trials in the whole experiment. A 10-min break was provided between the two tasks to avoid perception fatigue. The order of the two tasks was counterbalanced across the participants.

### Statistical Analysis

The data were analyzed using SPSS 23.0 (SPSS Inc., Chicago, IL, United States) with the significance level set at 0.05. The IPS and PPS were measured in centimeters. The average distances in each trial were calculated for further analysis. A separate analysis of variance (ANOVA) was conducted for each comfort distance and reachability distance task. A 2 × 8 × 2 ANOVA with participant’s gender (male, female) as the between factor and two within factors of direction (eight angles) and confederate’s gender (male, female) was used for IPS, whereas PPS was analyzed using a 2 × 5 × 2 ANOVA with a between factor (participant’s gender) and two within factors (direction and confederate’s gender). The Tukey *post hoc* test was used for *post hoc* comparisons of the significant effects. To compare the differences between the comfort distance and reachability distance in the five directions in front of the body, a 2 (participant’s gender) × 5 (direction) × 2 (confederate’s gender) × 2 (comfort/reachability task) ANOVA was applied. Partial η^2^ was calculated to indicate the magnitude of the significant effects.

## Results

### Results for Interpersonal Space

**FIGURE 2:**
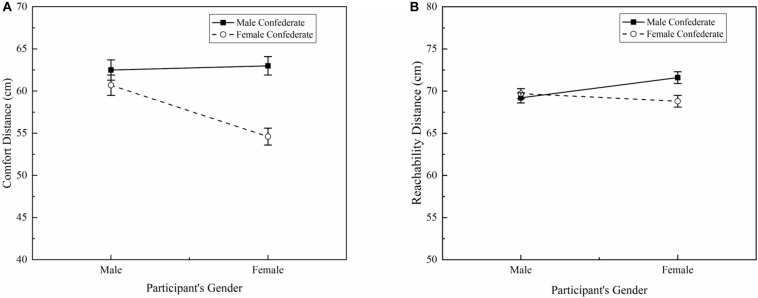
The effect of participant’s gender × confederate’s gender on IPS **(A)** and PPS **(B)**.

The significant ANOVA effects are shown in Table 1. Analysis of variance found a significant effect of participant’s gender [*F*(1, 1,056) = 12.12, *p* < 0.01, η^2^ = 0.11]. The male participants (mean = 61.6 cm, SD = 18.7 cm) maintained greater distances than female participants (mean = 58.8 cm, SD = 19.0 cm). A significant effect of confederate’s gender was found [*F*(1, 1,056) = 29.84, *p* < 0.001, η^2^ = 0.28]. A significantly larger distance was obtained when facing the male confederate (mean = 62.7 cm, SD = 19.7 cm) than the female confederate (mean = 57.6 cm, SD = 17.8 cm). Moreover, the effect of direction was significant [*F*(7, 1,056) = 80.24, *p* < 0.001, η^2^ = 0.39]. In the Tukey *post hoc* comparison, the distance in the front (0°) direction (mean = 73.9 cm, SD = 18.6 cm) was significantly larger than the other seven directions, whereas the distance in the rear (135°, 180°, 225°) was significantly closer than the other five directions. The eight directions were classified into three groups (all *p* < 0.05), as shown in Table 2. In addition, a significant interaction effect between participant’s gender and confederate’s gender was found [*F*(1, 1,056) = 13.27, *p* < 0.001, η^2^ = 0.13]. In Figure 2A, female participants maintained a significantly larger comfort distance from the male confederate than the female confederate (*p* < 0.001). However, the male participants showed no significant difference when facing the male or the female confederate. Other interaction terms of the ANOVA results were not significant.

**TABLE 1:** Significant ANOVA results and effect sizes for this experiment.

Effect	*F*	*df*	*p* value	η^2^
**Comfort distance**				
Participant’s gender	12.12	1	0.001	0.11
Confederate’s gender	29.84	1	0.000	0.28
Direction	94.64	7	0.000	0.39
Participant’s gender × confederate’s gender	13.27	1	0.000	0.13
**Reachability distance**				
Confederate’s gender	6.61	1	0.010	0.11
Direction	5.76	4	0.000	0.03
Participant’s gender × confederate’s gender	11.31	1	0.001	0.15
**Distance**				
Participant’s gender	8.54	1	0.004	0.06
Confederate’s gender	9.63	1	0.002	0.07
Direction	3.51	4	0.007	0.01
Direction × task	6.15	4	0.000	0.18
Confederate’s gender × task	30.83	1	0.000	0.22
Participant’s gender × confederate’s gender	25.21	1	0.000	0.18

**TABLE 2:** Means (SD) of interpersonal space (cm) results in the eight directions among genders.

Direction	0°	45°	90°	135°	180°	225°	270°	315°
Distance	73.9 (18.6)	68.6 (16.5)	68.4 (17.0)	45.0 (11.7)	44.2 (13.5)	47.6 (12.1)	67.1 (15.1)	66.8 (14.4)
Group^#^	A	B	B	C	C	C	B	B

*^#^Tukey range *post hoc* test results.*

### Results for Peripersonal Space

The ANOVA results showed a significant effect of confederate’s gender [*F*(1, 735) = 6.61, *p* < 0.05, η^2^ = 0.11]. Participants maintained a larger reachability distance when facing the male confederate (mean = 70.4 cm, SD = 9.0 cm) than the female confederate (mean = 69.3 cm, SD = 8.9 cm). No significant effect was found for participant’s gender (*p* > 0.05). Furthermore, a main effect of direction was found [*F*(4, 735) = 5.68, *p* < 0.001, η^2^ = 0.031]. The Tukey *post hoc* test results showed that the reachability distances in the five directions could be divided into two groups (Table 3). Participants displayed a larger distance on the right-hand side (mean = 72.7 cm, SD = 9.2 cm, *p* < 0.05) than in the other four directions. The participant’s gender and confederate’s gender showed a significant interaction effect [*F*(1, 735) = 11.31, *p* < 0.01, η^2^ = 0.13], as shown in Figure 2B. For the female participants, a larger reachability distance was found when facing the male confederate than the female (*p* < 0.05). No significant difference appeared in male participants. Other ANOVA interaction terms were not significant.

**TABLE 3:** Means (SD) of the peripersonal space (cm) in the five directions among genders.

Direction	0°	45°	90°	270°	315°
Distance	68.6 (9.2)	70.1 (8.1)	72.7 (9.2)	70.4 (9.2)	68.4 (8.5)
Group^#^	A	A	B	A	A

*^#^Tukey range *post hoc* test results.*

### Comparative Analysis of the Interpersonal and Peripersonal Spaces

**FIGURE 3:**
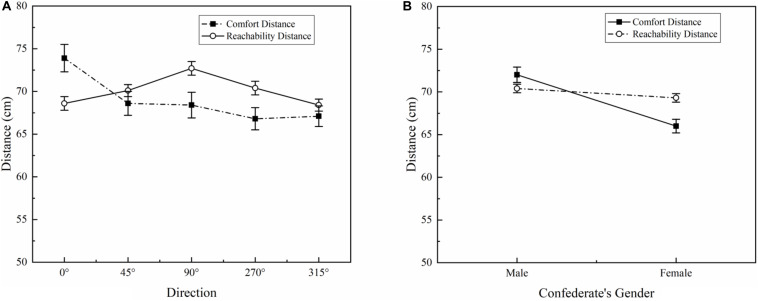
The IPS and PPS in different directions **(A)** and confederate’s genders **(B)**.

Analysis of variance found no significant difference between IPS (mean = 69.1 cm, SD = 16.5 cm) and PPS (mean = 70.0 cm, SD = 9.0 cm) (Table 1). A significant direction × task interaction appeared [*F*(4, 1,410) = 6.15, *p* < 0.001, η^2^ = 0.18], as illustrated in Figure 3A. In the front direction (0°), the comfort distance (mean = 73.9 cm, SD = 18.6 cm) was significantly larger than the reachability distance (mean = 68.6 cm, SD = 9.2 cm) (*p* < 0.05). Contrariwise, participants maintained a closer comfort distance than reachability distance on both the left (270°) and right (90°) sides (*p* < 0.05). In addition, confederate’s gender significantly interacted with task [*F*(1, 1,410) = 30.83, *p* < 0.001, η^2^ = 0.22], as can be seen in Figure 3B. When facing a male confederate, participants preferred a larger comfort distance than reachability distance (*p* < 0.01). Moreover, the comfort distance was significantly closer than the reachability distance when facing a female confederate (*p* < 0.001).

## Discussion

**FIGURE 4:**
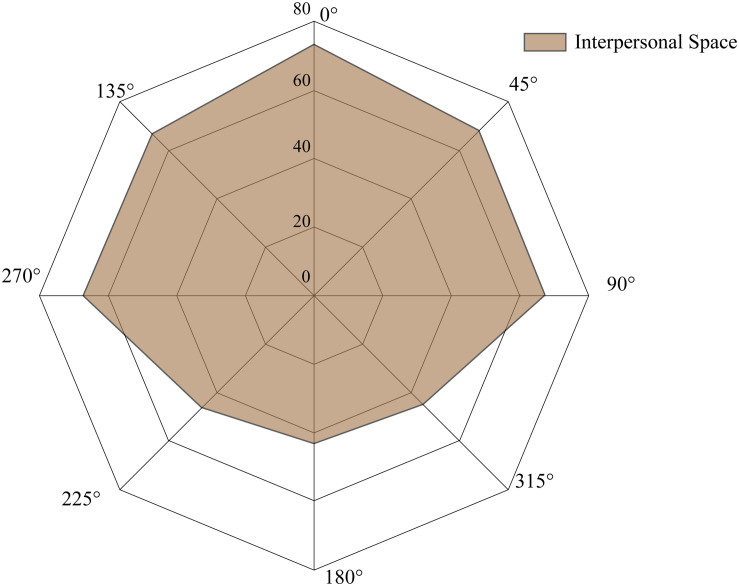
The shape of interpersonal space around the Chinese participants in eight directions among genders (unit in centimeters). The direction 0° identifies the front of participants.

This study found that the direction effect was significant in the IPS. The results showed that participants provided the largest comfort distance in front of their bodies, with the closest distance in the back and intermediate distances laterally. Thus, the shape of IPS around a Chinese person is non-circular (Figure 4). The results of this study were consistent with those of several other studies (Hayduk, 1981; Yang, 1988; Bailenson et al., 2003), but differed from the results of Hall (1966) and Hecht et al. (2019). Bailenson et al. (2003) reported that participants preferred a larger distance when they were in front of a virtual human than in the rear or side cases. Similarly, Hayduk (1981) suggested that the shape of IPS was non-circular, with declination from the largest in the front to the smallest in the rear. In contrast, Hecht et al. (2019) found the shape of the IPS to be circular, and no significant difference was found in the eight directions. These discrepancies could be attributed to different experimental methods. In Hecht et al. (2019), the active participants were required to choose a comfortable distance at which they can have a conversation with the passive participants. Hence, the appropriate talking distance could be relatively stable without varying by direction. In this study, the typical method of “stop distance” was applied, which better reflects the discomfort of the participants in a situation where their comfort zone was intruded upon (Dosey and Meisels, 1969; Hayduk, 1983; Adams and Zuckerman, 1991; Gessaroli et al., 2013). This research first classified the IPS of Chinese participants in eight directions into three groups: the front (0°), the lateral area (45°, 90°, 270°, 315°), and the rear area (135°, 180°, 225°). A possible reason for this finding is that the participants felt greater threat and pressure from a directly opposing impact. In addition, there was a low level of intimacy and less sensitivity when the participants were approached from behind. It was easier for participants to feel the confederate’s approach from front directions (such as 45°) than rear ones (such as 180°). Hence, the interpersonal distance in the rear area was shorter than in the front and lateral areas (Hayduk, 1981).

In comparison with other cultures, the IPS of Chinese people in the front (73.9 cm) was closer than those of the Japanese (134.6 cm) and German subjects (110.5 cm) measured in Sicorello et al. (2019). The comfort distances of Caucasian subjects (100.5 cm) reported by Hecht et al. (2019) were also larger than those found in the present study. These results indicate that although China is a non-contact culture, the IPS in the Chinese cultural background is closer than those of Caucasians and Japanese. We know that China has a huge population, leading to a very crowded social environment in public spaces. Chinese people have to maintain a smaller distance between themselves and others in daily life, especially in subways and bus stations. The findings of this study imply that IPS is influenced by the culture and the environment.

Regarding gender, the participants kept a larger IPS with male confederates than female ones. This finding was consistent with previous studies (Iachini et al., 2014, 2016). Female participants preferred a significantly shorter comfort distance when facing a female than a male confederate. For male participants, the comfort distances from male and female confederates were similar, and both were larger than in female dyads. These findings are contrary to Evans and Howard (1973), who found that mixed-gender dyads interacted more closely than female dyads. Moreover, Hecht et al. (2019) stated that there was no difference in the IPS between mixed-gender dyads and same-gender dyads. A possible explanation for the inconsistent results is cultural differences. In traditional Chinese culture, people were more conservative in opposite-sex relationships. Chinese participants felt sensitive and shy when an unfamiliar confederate of the opposite sex approached (Yang, 1988). Hence, longer IPS was maintained in the Chinese mixed-gender dyads to maintain comfort than in same-gender dyads.

This study found that Chinese participants’ PPS in the five directions showed a larger distance on the right-hand side (direction of 90°) than in the other four directions. This finding might be attributed to all participants being right-handed. The stronger capability of the dominant hand would increase the reachable distance (Linkenauger et al., 2009). The male participants displayed a similar reachability distance as the female participants, which corroborates Iachini et al. (2014). Similar to the comfort distance, participants maintained a greater reachability distance from a male confederate than from a female confederate. This finding was in agreement with Iachini et al. (2014). Additionally, female participants maintained a larger reachability distance from a male confederate than a female confederate. Male participants showed no significant differences between genders. These findings are similar to the interpersonal distance results identified in the present study, which indicates that both IPS and PPS are similarly sensitive to certain social aspects such as gender (Iachini et al., 2016).

The relationship between IPS and PPS has been discussed extensively in the proxemic and neurocognitive literature. Iachini et al. (2014) pointed out that IPS and PPS were similar when the participants actively approached the confederate but differed when participants were passively approached. Iachini et al. (2016) found that participants maintained larger IPS and PPS when facing male confederates than female confederates. The present research further investigated the relationships between the two spaces for Chinese participants in the five selected directions. The effects of task (IPS and PPS) showed no significant differences, but task interacted significantly with direction. The IPS was significantly larger than the PPS in the front direction (0°), whereas the IPSs on the right (90°) and left (270°) sides were closer than the PPSs. That means when facing confederates approaching from the front direction, a stronger feeling of insecurity and pressure was triggered. This might explain why a significant difference was found in the front direction between IPS and PPS, whereby participants stopped the confederates before they entered the reachable range because of feelings of discomfort. Laterally (90° and 270°), participants preferred a closer comfort distance than reachability distance, indicating that participants allowed others to walk into their reachable range in the lateral areas of their bodies. The IPS of participants was larger than the PPS when facing a male confederate, but the opposite was true when facing a female confederate. These findings could be related to a higher acceptability of females; participants showed a higher tolerance for female confederate proximity in the front, even within the reachable range (Cui, 2019).

**FIGURE 5:**
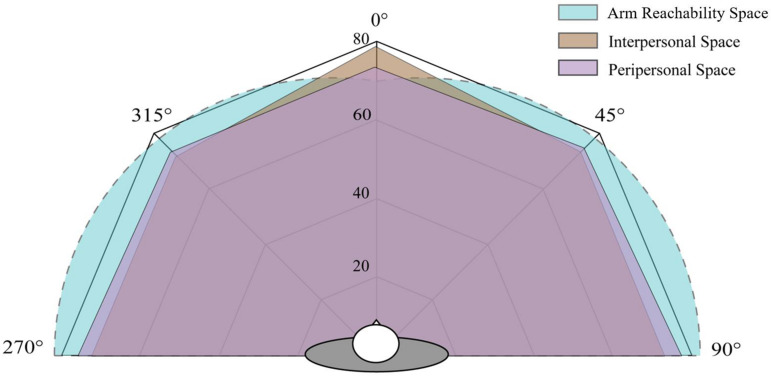
The arm reachability space, interpersonal space, and peripersonal space of Chinese participants in the five directions (unit in centimeters).

The PPS is regarded as a multisensory visuomotor space in which individuals can touch objects (Rizzolatti et al., 1997, 2002; Grefkes and Fink, 2005). That is, the boundary of this area is the interface of the area where individuals are able to touch objects with their hands (Brozzoli et al., 2013; Teneggi et al., 2013; Iachini et al., 2016). The typical method for measuring this distance is for participants to stop approaching confederates when the participants feel that they could touch them (Iachini et al., 2014). In order to further explore the relationships between the participants’ arm length and the space they feel they could reach, the average arm length of participants was compared to the IPS and PPS. Figure 5 illustrates the space of arm length, IPS, and PPS when considering the participants’ shoulder breadth (37.4 cm) and the bigonial width (11.6 cm). It is interesting to note that participants tended to overestimate their reachability distance in the front (arm length: 70.8 cm; reachability distance: 74.4 cm), whereas the distance participants could reach laterally (45°, 90°, 270°, 315°) was underestimated. These findings suggest that individuals ignored the breadth of their shoulders. Participants might think of themselves as a particle without taking into account the fact that the lateral reachable distance is larger than in the front because of their shoulder breadth. Of the three spaces, two of them, IPS and PPS, pertain to psychological perception; the third, the arm length space, is related to physical ability. These spaces ranged from 65 to 80 cm around the individuals, which falls within the range of personal distance reported by Hall (1966).

The findings of IPS and PPS in this study could be taken into consideration in environmental design. For example, when arranging the placement of tables and chairs in a space, the distance between the seats can be set according to the IPS boundary so as to maximize the space usage in order to ensure the users’ comfort. The PPS boundary and the arm length space can also guide human–machine interface design. The buttons on the interface should be placed in positions where users feel they can easily reach the buttons by considering the PPS distances in different directions.

## Conclusion

This study investigated the IPS and PPS of Chinese participants in different directions and gender groups. The results indicate that the IPS of Chinese people is non-circular, with the distances in eight directions classified into three groups (the largest in the front and the closest in the rear). The PPS on the right side was larger than in the other directions. Participants maintained a larger IPS and PPS with a male confederate than a female confederate. Thus, the two kinds of spaces were similarly sensitive to social stimuli in some cases. Additionally, the confederate’s proximity in the front constitutes greater intrusion, so the IPS in front was beyond the reachability range. On the right and left sides, a closer comfort distance was allowed even within the reachable area. When facing a male confederate, a larger IPS was shown than PPS, whereas the IPS was closer than the PPS when facing a female confederate. The findings of this study strengthen the idea that the space surrounding individuals is regulated not only by the perception of external social valance but also the motor ability. It was interesting to note that participants overestimated their reachability distance in the front, while underestimating their reachability distance laterally. The findings in this study contribute to an understanding of the motor nature of the space near human bodies. Perception and action could collaborate in dealing with the space around the human body. The IPS and PPS boundaries could be applied in environmental design, space utilization, and social interaction.

## Data Availability Statement

The datasets generated for this study are available on request to the corresponding author.

## Ethics Statement

The experimental procedures were approved by the Institutional Ethics Committee of South China University of Technology, and the study was performed in accordance with the Declaration of Helsinky (2013). The participants provided their written informed consent to participate in this study.

## Author Contributions

Y-CL contributed in building up the idea, experiment, proofreading and lead the project. XY and WX conducted the experiment and performed the statistical analysis. XY and Y-CL wrote the first draft of the manuscript. WX wrote portions of the manuscript.

## Conflict of Interest

The authors declare that the research was conducted in the absence of any commercial or financial relationships that could be construed as a potential conflict of interest.
